# Factors influencing pharmaceutical companies’ decisions to pursue compassionate use programs in the EU: a qualitative study in The Netherlands

**DOI:** 10.1080/20523211.2025.2605391

**Published:** 2026-01-06

**Authors:** Aimée Timmerman, Nienke Rodenhuis, Lucia Marie Albertine Crane-van Opstal, Anthonius de Boer, Leon Bongers, Anna Maria Gerdina Pasmooij

**Affiliations:** a Dutch Medicines Evaluation Board, Utrecht, The Netherlands; b Dutch Health and Youth Care Inspectorate, Utrecht, The Netherlands; c Division of Pharmacoepidemiology and Clinical Pharmacology, Utrecht Institute for Pharmaceutical Sciences, Utrecht University, Utrecht, The Netherlands

**Keywords:** Compassionate use, named patient, early access, medicines, unmet need

## Abstract

**Background:**

Access to unauthorized medicines in the EU is legally restricted, except in certain cases such as clinical trials, magistral preparations, hospital exemptions, and early access programs, including compassionate use programs (CUPs) and named patient use (NPU). CUPs, regulated under Article 83 of Regulation (EC) No 726/2004, are intended for a group of patients with an unmet medical need. Despite this EU-wide regulation, the implementation of CUPs varies among member states, and the factors driving pharmaceutical companies to pursue them are poorly understood.

**Methods:**

This study conducted semi-structured interviews with pharmaceutical companies that had applied for CUPs in the Netherlands, as well as those with potentially eligible medicines that had not pursued CUPs. The interviews explored the decision-making processes and factors influencing CUP applications. Transcripts were analyzed using Atlas.ti software, with coding categories derived from the interview guide and emerging themes.

**Results:**

Ten interviews were conducted. Factors influencing CUP applications were classified into four categories: regulatory, medical, operational, and financial. Regulatory factors included recommendations from the Health and Youth Care Inspectorate (IGJ) and European Medicines Agency (EMA), concerns about post-marketing authorization uncertainties, and timelines for CUP approval. Medical factors involved unmet medical needs, patient numbers, and the alignment of CUP indications with authorized indications. Operational factors included prior experience with CUPs, supply availability, and the appeal of NPU due to faster approval times. Financial factors centered on reimbursement expectations and decisions by company headquarters on the free provision of medicines.

**Conclusion:**

The decision to pursue CUPs is influenced by multiple factors, with regulatory uncertainties and operational complexities playing significant roles. Improving clarity concerning CUP regulations, particularly data collection and the post-marketing phase, could encourage more pharmaceutical companies to apply for CUPs, which would provide patients with earlier access to potentially promising treatments.

## 1. Background

Access to medicines is vital in promoting individuals’ health and well-being across all age groups. However, medicines must be authorized before they can be made available for daily patient use. Per EU law, unauthorized medicines can only be prescribed in specific situations, such as magistral preparations, clinical trials and hospital exemptions, and alternative pathways, including compassionate use programs (CUPs) and named patient use (NPU). CUPs and NPU, also referred to as early access programs, offer avenues for accessing unauthorized medicines, particularly in cases of an unmet medical need.

CUPs originated in the EU due to a historical imperative to expedite access to treatments during public health crises such as the HIV epidemic (Wagner et al., 2020). The regulatory framework was established in Article 83 of Regulation (EC) No 726/2004 at the European level. In 2007, the EU Guideline on Compassionate Use was adopted (Committee for Medicinal Products for Human Use [CHMP], 2007). However, the implementation of CUPs remains contingent on individual member states’ discretion, which reflects the diverse legal and regulatory landscapes and procedures (Rahbari & Rahbari, 2011). CUPs specifically involve a cohort (i.e. patient group) to which an authorized medicine can be made available. Pharmaceutical companies can request approval for a CUP from national competent authorities to provide these medicines to patients in need before obtaining marketing authorization. The National Competent Authority (NCA) subsequently assesses whether the CUP criteria have been met. For example, this assessment in the Netherlands, performed by the Dutch Medicines Evaluation Board (MEB), is based on the severity of the disease, the unavailability of authorized alternatives, and whether a positive benefit-risk ratio is likely. The CUP officially ends when the marketing authorization is granted. Subsequently, pharmaceutical companies must ensure that the medicine remains available until it reaches the market, including during the reimbursement phase.

In contrast to CUPs approved for a cohort, NPU is limited to individual patients. Similar to CUPs, differences exist between European member states’ NPU protocols. In the Netherlands, the Health and Youth Care Inspectorate (IGJ) is the competent authority for an NPU request. If, according to a physician, treatment is impossible with the currently authorized medicines, permission can be requested from the IGJ to provide a medicine without marketing authorization. These medicines may concern unauthorized medicines, including investigational and authorized medicines in the EU or countries with whom the EU has mutual recognition agreements (MRAs). The criteria for providing such medicines are based on the patient’s medical need, as deemed by the prescribing physician. To obtain permission from the IGJ, the physician must explicitly state why the patient cannot be treated with medicines authorized in the Netherlands (Health and Youth Care Inspectorate, 2024). Table 1 provides a comparison between a CUP and NPU.

**Table 1.** Compassionate use program (CUP) vs. named patient use (NPU) in the Netherlands.

	CUP	NPU
EU Legislation	Article 83 of Regulation (EC) No 726/2004	Article 5(1) of Directive 2001/83/EC
NL Legislation	Article 3.18 of the Dutch Medicines Act Regulations	Article 3.17 of the Dutch Medicines Act Regulations
Purpose	To provide access to unauthorized medicines to patients with life-threatening conditions with no registered alternatives	To provide access to unauthorized medicines to patients with no registered alternatives and an unmet medical need
Initiator	Pharmaceutical company	Physician, pharmacy, wholesaler, or pharmaceutical company
Eligibility	Group of patients (cohort)	Individual patient
Regulatory body	MEB	IGJ
Benefit-risk assessment	Assessed by the MEB	No benefit-risk assessment by the IGJ
Duration	Valid until marketing authorization	Valid for 1 year; an extension is possible

While the benefits and risks of these early access programs have been discussed in the literature (Patil, 2016; Tarantola et al., 2023), the factors driving pharmaceutical companies to pursue CUPs are poorly understood. Therefore, we investigated the decision-making process of pharmaceutical companies applying for CUPs and the factors influencing their decisions.

## 2. Methods

Semi-structured interviews were conducted with pharmaceutical companies operating in the Netherlands. The interviews contained open-ended questions and were conducted between April 17 and May 11, 2023, in Dutch. An interview guide (see Supplemental Material) was utilized that focused on decision-making processes regarding CUPs within the pharmaceutical company and the motives to apply (or not apply) for one. Prior to each interview, the interviewees provided informed consent. The first interview was conducted by two researchers (L.B. and A.T.) and served as a pilot interview. The research team conducted an evaluation after the interview. Thereafter, nine more interviews occurred. For the interviews, ethical approval was obtained from the Research Ethics Committee (CETO) of the Faculty of Arts, University of Groningen with protocol number ID 102394219.

### 2.1. Interviews

We aimed to interview pharmaceutical companies with previously approved CUPs in the Netherlands and those that had not applied for CUPs, but were applicants for marketing authorization at the EMA for medicines that could have potentially applied for a CUP at the NCA in the Netherlands (i.e. the MEB). This division was chosen to gain a broad perspective on the reasons for applying for a CUP. The contacts responsible for the marketing authorization application were approached; they either participated in the interview or directed us to the appropriate experts based on their expertise.

A list was compiled of the pharmaceutical companies that had previously received approval for their CUPs by the MEB in 2010–2022. Six companies from this list with some of the highest number of CUP requests were chosen for interviews due to their extensive familiarity with CUPs in the Netherlands. Additionally, five companies were selected for interviews based on the presence of eligible CUP medicines that had not been pursued. For this second selection, a list of medicines was compiled based on marketing authorization applications submitted through the centralized procedure at the EMA between January 1, 2020, and December 31, 2022. Medicines indicated for the treatment of serious or life-threatening conditions without existing authorized alternatives were considered eligible for a CUP. Consultation was sought with MEB clinical assessors with expertise in the disease area of each medicine to verify the eligibility of the identified CUP medicines. In total, 16 medicines from 12 pharmaceutical companies were identified as eligible for CUP. Of these 12 companies, five companies had never previously applied for CUPs at the MEB and maintained an office in the Netherlands. These five companies were invited to participate in the interviews.

### 2.2. Data analysis

Interviews were recorded using the Dictaphone application on a smartphone, and transcripts were generated using Transkriptor software and manually reviewed for errors. The transcripts were then subjected to coding to establish categories and connections between them. These categories were initially derived from the interview guide (using deductive coding) but were further refined based on emerging themes from the interviews (using inductive coding). Data analysis was conducted using Atlas.ti 23.1.2 software for Mac. All transcript coding was performed by one researcher (A.T.). The codes were discussed with two researchers (L.B. and A.P.) to enhance the reliability of the coding process and maintain data quality. Additionally, all data were anonymized to protect interviewee confidentiality.

## 3. Results

This study systematically categorized and analyzed the factors impacting the pursuit of CUPs by pharmaceutical companies in the Netherlands. Between January 1, 2010, and December 31, 2022, 69 CUP requests were submitted in the Netherlands, with 36 granted. This ratio accounted for just under three CUPs granted per year.

Of the 11 pharmaceutical companies invited for an interview, one declined to participate. As a result, five companies with CUP medicines and five companies with eligible CUP medicines were interviewed. Among the five companies with CUP medicines, four had previous CUP applications rejected. The company contacts for this study were selected based on their experience with CUPs within their pharmaceutical company. In some cases, initial contacts referred us to the colleague most familiar with CUPs. Half the interviewees were employed in medical functions, while the other half held positions in regulatory roles. An overview of the results with quotes appears in Table 2.

**Table 2.** Overview of interview results. (EMA: European Medicines Agency, CGR: Code for Pharmaceutical Advertising, CUP: Compassionate Use Program, IGJ: Health and Youth Care Inspectorate, NPU: Named Patient Use).

Main Theme	Sub themes	Key considerations	Illustrative quotes	Number of interviewees (n = 10)
Factors that influence whether a pharmaceutical company applies for a CUP	Regulatory factors	CUP recommendation from IGJ or EMA	‘We started off that way, and at some point, we had around 8 or 10 patients or so. Then the IGJ indicated, “you really need to submit a CUP application for a cohort now.”’ (quote 1)	4
		Prospects of marketing authorization	‘Is there potential to have registration for the product at all, is a very crucial question.’ (quote 2)	6
			‪‘Often, the reasons are that there is not enough clinical evidence, for instance, that as a company, we don’t feel comfortable providing an experimental, unauthorized product, especially for indications that have not been investigated.’ (quote 3)	1
		Fear of complication during CUP	‘But what if something goes wrong in the CUP? For instance, if the patient dies during the CUP. Does it have a negative impact on that EMA decision?’ (quote 4)	1
		Uncertainties after marketing authorization	‘Yes, but that is now a bit unclear, I think, in the new policy document. Yes, it seems more complicated for companies to initiate a CUP because you’re not exactly sure how far you should go with your CUP program. It’s always a difficult consideration because you just want to continue treating your patients.’ (quote 5)	7
			‪‘It’s a gray area, right? Let’s establish that upfront, so there is no legal formal basis, right? And there is also no formal process for it, where we always adhere to.’ (quote 6)	2
			‘You’re not allowed to give away, then we enter the realm of advertising and pharmaceutical advertising regulations (CGR), and that’s not allowed because it can influence someone in a way that they continue to prescribe. Yes, that’s a significant problem in that aspect.’ (quote 7)	6
	Medical factors	Unmet medical need	‘If there is a need, we try to see what we can do for a patient.’ (quote 8)	8
			‘It’s usually the doctors who recognize the necessity and have patients in need of treatment.’ (quote 9)	8
			‘These diseases are so urgent we are faced with, so you go to any lengths to make it available.’ (quote 10)	1
		Number of patients	‘Whether you expect many or few patients is taken into consideration.’ (quote 11)	8
			‘I think you would consider applying for a CUP as the numbers of patients increase. Because then it’s, so to speak, worth your time to invest in it.’ (quote 12)	8
		Chronic conditions	‘What I also see is that there is a difference in an oncological treatment or, well, a defined number of cycles someone could receive versus a chronic treatment. Because in that case, you have to think very carefully, what are we committing to?’ (quote 13)	3
		Inclusion criteria	‘There is a risk with reasonably broad inclusion of patients in a CUP.’ (quote 14)	1
	Operational factors	Speed of application process	‘It would be encouraging if the timeline for a CUP is shorter.’ (quote 15)	5
			‘We also can’t wait for days or weeks because by then, the patient may no longer need it.’ (Quote 16)	5
		Standardization and global protocols	‘And we knew from the headquarters that they had prepared the necessary documents. Such a protocol, etc., was already in place, and we also expected more than just one or two patients, so those considerations were taken into account.’ (quote 17)	3
		Product supply and availability	‘Can it be delivered at all? That is also crucial.’ (quote 18)	4
		Experience with CUP	‘Well, in the past, we didn’t have much experience with applying for and getting approval for a CUP, so the enthusiasm wasn’t immediately high.’ (quote 19)	4
			‘Yes, we’re going to submit a new one, I happen to know it’s coming from the hematology department. They heard about my experiences, so they were enthusiastic, and they are going to give it a try as well.’ (quote 20)	1
	Financial factors	Prospect of reimbursement	‘There must always be prospects for registration and reimbursement.’ (quote 21)	5
		No free product supply headquarters	‘It plays a significant role, at least for us. Our headquarters has decided not to go for free provision anymore. So, doctors do come to us for named patient use or CUP, and we can provide that, but it has to be paid for, and that doesn’t fall under any reimbursement structure. So, as a result, the patient does not get the medication.’ (quote 22)	2
CUP vs NPU	Confusion in terminology	The different terms can cause confusion	‘There are a lot of terms that can be confusing.’ (quote 23)	7
	Choice CUP or NPU	No reasons to apply for CUP	’The site manages the approvals itself and the IGJ. Yes, then you can also handle that, right? Yes, we had all the things ready. Suppose the IGJ now says, “well, guys, you’ve already treated so many patients, you have to move on to a CUP.” No, and then we thought, well, then there’s actually no reason for us to apply for a CUP. One reason could be that it’s on the website, right?’ (quote 24)	1
		Cohort definition	‘A specific number instead of the term “cohort”. That would be nice because, yes, the indications we have, they are just relatively small patient populations. So if you know it’s going to be around 10 patients, for example, then automatically transitioning to a CUP could be possible. Or starting with supply based on a doctor’s statement.’ (quote 25)	1
		Preference NPU	‘Yes, our preference really leans towards named patient use.’ (quote 26)	4
	NPU cohort	Applying for a NPU cohort	‘We received a cohort from the IGJ, so the CUP did not materialize either.’ (quote 27)	3
	Use of NPU after marketing authorization	NPU cohort after marketing authorization	‘The product was authorized but not yet reimbursed. Legally, we are not allowed to start a CUP in that case. However, we are allowed to start named patient use. Named patient use can be initiated from a hospital, so it is not intended per patient but for a group.’ (quote 28)	1
Suggested improvements	Data collection	The desire the collect data in a CUP	‘And, it would certainly help if we could have an opportunity to collect more data than just relying on doctors reporting adverse events. Because when we talk about that data in your local market and how important it is, and if we discuss cost-effectiveness, having that capability would be beneficial.’ (quote 29)	7
			‘Yes, if you can already gather real-world evidence or real-world data during your CUP, that could be very valuable.’ (quote 30)	1
			‘Well, if you can collect data, then I would definitely have a preference for a CUP.’ (quote 31)	1
	CUP application	Clear application process	‘I found it quite clear what they actually wanted from you. So, on the website, it is very clearly described, I thought, what you had to submit. So, I found all of that quite clear.’ (quote 32)	7
		More transparency and the application form on the MEB website	‘That could use a bit more transparency because it might make it more non-committal to look for information. It almost feels like a barrier to request that application form via email, whereas if you could have downloaded it at some point, you could say, oh, do we actually have all this? And maybe you could have tried it much sooner.’ (quote 33)	2
			‘For example right? How does this work, how do I do this? Just provide information to a person, you know? And as far as I’m concerned, you can also automate a lot, right? It doesn’t have to be an actual physical person. But as, I said, this is not our daily work.’ (quote 34)	2
		Contact person who can help with the CUP application	‘What I found difficult is that you don’t really have a kind of contact person where you can inquire about the status. You do get a reference number to refer to, of course, but you can’t just pick up the phone and ask, “hey, how’s it going?” That was challenging for me. It feels a bit like a black box where you put something in, and the answer comes out at some point. So yes, I found that challenging. I hope it gets approved as soon as possible. So you’re a bit on edge, and you’re thinking, when will there be white smoke?’ (quote 35)	2

**Figure 1.**
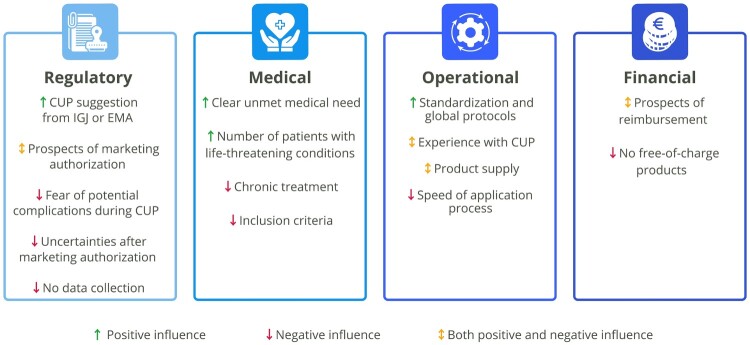
Factors influencing whether a pharmaceutical company applies for a CUP in the Netherlands, structured in four categories. (CUP: Compassionate Use Program, EMA: European Medicines Agency, IGJ: Health and Youth Care Inspectorate).

The interviews were coded to identify specific factors influencing CUP pursuit. We classified these factors into four distinct categories: regulatory, medical, operational, and financial. A detailed analysis incorporating these factors is presented in the subsequent sections. An overview of these factors is shown in Figure 1.

### 3.1. Regulatory factors

Within the regulatory domain, the interviewees referenced the influence of recommendations from the IGJ and EMA. When faced with multiple NPU applications, the IGJ suggested considering a CUP application at the MEB (Quote 1). Another regulatory factor mentioned was the recommendation from the Committee for Medicinal Products for Human Use (CHMP) of the EMA. One interviewee reported receiving a recommendation from the CHMP for a CUP in Europe and subsequently applied for a CUP in the Netherlands based on this recommendation:

We started like that, and at some point, we had around eight or ten patients. And then the IGJ indicated, ‘You really need to submit a CUP application for a cohort now’. (Quote 1)

Several interviewees highlighted the prospects of EMA authorization for the medicine as essential. The interviewees emphasized the need for assurance that the medicine would eventually receive marketing authorization from the EMA (Quote 2). Moreover, one interviewee explained that the company had not applied for a CUP due to a lack of clinical evidence and confidence in making an unauthorized medicine available to patients (Quote 3). One interviewee expressed concerns about potential adverse reactions that could arise during the CUP and the implications on the EMA decision (Quote 4):

But what if something goes wrong in the CUP? Suppose the patient dies in the CUP. Would that have a negative impact on that EMA decision?

Furthermore, most interviewees perceived risks during the period following marketing authorization and extending to the reimbursement phase (Quote 5) due to a lack of clear guidelines (Quote 6). Concerns were voiced regarding compliance with the Code of Conduct for Pharmaceutical Advertising (CGR), particularly considering the CUP policy changes in the Netherlands since 2023, since the legal basis for a CUP expires after marketing authorization (Quotes 5 and 7).

Notably, a preference for the NPU route emerged, driven by its streamlined process, one-year validity, and the avoidance of post-marketing authorization uncertainties (Quote 26). This preference was also due to the interviewees being more familiar with NPU than with CUPs.

The timeframe for reviewing CUP applications at the MEB emerged as an important factor for pharmaceutical companies when considering whether to apply. Half the interviewees emphasized that patients with urgent medical needs could not afford to wait the standard six-week review period at the MEB (Quote 16). The lengthy CUP approval process often led companies to opt for NPU through the IGJ instead. The official time for NPU is eight weeks, but the interviewees acknowledged that it is often granted within a few days, which further strengthened its attractiveness as a practical alternative.

It would be beneficial if the timeline of a CUP is shorter. (Quote 15)

Most interviewees highlighted the wish for more comprehensive data collection beyond adverse events (e.g. for efficacy) as an important factor in improving CUPs (Quote 29). They stressed that collecting additional data would both aid the assessment of efficacy and cost-effectiveness of treatments. The interviewees recognized the value of real-world data collection during CUPs as highly valuable for reimbursement purposes (Quote 30). However, they acknowledged the complexities involved in implementing data collection within CUPs, due to uncertainties concerning rules on data collection.

Yes, if you can collect real-world evidence or real-world data during your CUP, that can be very valuable. (Quote 30)

### 3.2. Medical factors

One of the medical considerations for making the medicine available before marketing authorization involved recognizing an unmet medical need and responding to the demand expressed by healthcare professionals (Quote 9). The importance of medical necessity varied depending on the indication considered. The severity and urgency of the condition were highlighted as critical factors in determining the medical need for the treatment. The ethical obligation to treat seriously ill patients was also mentioned as a driving force behind the decision to apply for a CUP:

These diseases that we are faced with are so urgent that you go to any lengths to make it available. (Quote 10)

Most interviewees identified the number of patients as another critical factor. The pharmaceutical companies aimed to ensure that a sufficient number of patients could benefit from the medicine. Thus, when patient numbers were limited, the preferred route for making the medicine available was through NPU (Quote 12).

Three interviewees also highlighted the challenges associated with chronic diseases compared to oncological and other time-limited treatments (Quote 13). They expressed concerns about what would happen when a CUP ended, including the decision-making process for continued treatment and the responsibility for providing the medicine for free. While primary a medical factor, this factor also included regulatory implications, particularly as the commitment to providing ongoing treatment for chronic conditions was perceived as more complex and required careful consideration (Quote 13). An additional point of concern, highlighted by one interviewee, was the potential inconsistency between the scope of the CUP indication and the marketing authorization indication (Quote 14). The interviewee feared the CUP label might have a broader scope than the marketing authorization label. While primary a medical factor, this factor also included regulatory implications, since the discrepancy between the CUP label and marketing authorization label could result in the provision of the medicine, free of charge, to patients who did not fall within the approved label for an extended period, potentially for the rest of their lives.

### 3.3. Operational factors

Five interviewees acknowledged the value of global protocols established by the pharmaceutical company’s headquarters for CUPs (Quote 17). These protocols simplified the initiation of CUPs in different countries, with local adaptations made to adhere to regional regulations. Additionally, the headquarters were noted as often hiring a contract research organization (CRO) to facilitate the implementation of the CUP:

And we knew from the headquarters that they had prepared the necessary documents. They had already developed a protocol and other relevant materials, and we anticipated more than just one or two patients, so we took those considerations into account as well. (Quote 17)

Some interviewees pointed out that prior experience with CUPs enhanced the ease of the process. When colleagues had positive experiences with CUPs, it lowered the barrier for others in the company to submit another CUP compared to when no prior experience existed (Quote 20). Conversely, one interviewee mentioned having limited CUP experience, which influenced not pursuing one because nobody knew how the process worked (Quote 19). The diverse terminology, such as early access programs, CUP, and NPU, was especially confusing, which led some to prefer NPU over a CUP (Quote 18).

The medicine supply was identified as an essential factor for initiating a CUP. Four interviewees emphasized that without sufficient supply, starting a CUP was unfeasible (Quote 18). Thus, pharmaceutical companies must carefully consider their production capacity and available stock, as they must balance the demand for clinical trials and CUPs. Despite pressing unmet medical needs, supply limitations may result in the inability to launch CUPs.

### 3.4. Financial factors

A crucial financial factor mentioned was the expectation of reimbursement for the medicine and the timing of its availability. One interviewee expressed uncertainty regarding reimbursement negotiations: since the patients already receive the medicine, this may slow down the reimbursement phase. Hence, the risks associated with the medicine’s reimbursement were a considerable concern. The shift in reimbursement practices and the potential financial burden over an extended period influenced the risk assessment and decision-making process regarding CUPs (Quote 21). Additionally, one interviewee noted that in situations where a medicine was authorized but not yet reimbursed, the pharmaceutical company also applied for NPU (Quote 28).

Decisions made by the pharmaceutical company headquarters regarding the provision of free medicines significantly impacted CUPs. Two interviewees highlighted that their respective company headquarters had decided to discontinue providing medicines free of charge. Consequently, these companies opted not to pursue CUPs, so patients could no longer receive the medicines at no cost.

It plays a very significant role, at least for us. Our headquarters has decided to discontinue providing free distribution. As a result, physicians now approach us for named patient use or CUPs. While we can supply the products through these programs, it comes at a cost, which does not fall under any reimbursement structure. Therefore, the consequence is that the patient does not receive the product. (Quote 22)

## 4. Discussion

This study explored the factors driving pharmaceutical companies to apply for a CUP, which were divided into four categories: regulatory, medical, operational, and financial.

**Regulatory factors** concerned the IGJ/EMA recommendations, prospects of marketing authorization, fear of potential complications during CUPs, uncertainties after marketing authorization, and uncertainties concerning rules on data collection. Of these regulatory factors, the latter two were considered the most important.

The interviewees expressed the desire for more comprehensive data collection within CUPs beyond just adverse events. Pharmaceutical companies considered this approach highly valuable. While the primary aim of CUPs is to provide treatment rather than to conduct research, several studies, including those by Berger et al. (2017) and Polak et al. (2022), have demonstrated that CUPs can generate valuable real-world evidence. This call for enhanced data collection during CUPs aligns with the EMA’s increasing focus on the potential of real-world data in regulatory decision-making. Although promising, to optimize and facilitate the increasing use of real-world data besides post-authorization safety data, Bakker et al. (2023) indicated that specific guidance is required to ensure that the data collected are fit for purpose in various regulatory contexts. EMA publications, such as the report on experiences gained with regulator-led studies from September 2021 to February 2023, can further strengthen the real-world evidence framework (European Medicines Agency, 2023). To fully realize this potential, harmonizing data collection across CUPs in the EU is essential. This requires collaboration among pharmaceutical companies, patient organizations, healthcare providers and regulatory authorities to standardize data collections methods. Barriers include inconsistent data recording practices and national regulatory differences. Future studies should investigate systematic methodologies for CUP data collection, particularly in support of real-world evidence integration into regulatory decision-making (Aliu et al., 2022).

The other significant regulatory factor highlighted by the interviewees concerned the transition period between marketing authorization and reimbursement. A lack of clear guidance governs this phase (i.e. where the CUP officially ends at registration of the medicine and the period until a decision on reimbursement has been made) because the medicine has already been made available through the CUP. Concerns were further raised about providing medicine at no charge (i.e. a **financial factor**) during this period and adherence to the rules on pharmaceutical advertising in the CGR code in the Netherlands. This issue echoed concerns noted in Patil’s (2016) white paper, where providing medicine for free was feared to be perceived as an attempt to promote unauthorized medicines. To address this regulatory gap, establishing clear guidelines by regulatory authorities for the phase after registration of the medicine is essential.

**Medical factors** entail determining a clear unmet medical need, whether the CUP indication is the same as the registration indication, the predictability of the number of patients, and the duration of treatment during the program. Most interviewees emphasized the importance of estimating the expected patient volume, one of the key medical factors that significantly influenced the decision between a CUP and NPU. Notably, the interviewees expressed uncertainty about where the line between these two programs should be drawn (e.g. at 10, 50, or even 100 patients). Moreover, pharmaceutical companies were noted as typically waiting for physician requests for the medicine, so the absence of these requests deters CUP pursuit. This practice aligns with the literature that states that in academic hospitals, physicians are expected to seek promising new treatment options (Bunnik & Aarts, 2021). The responsibility of informing patients about investigational treatment options is seen as part of the role of an academic medical specialist. However, some physicians may be reluctant to pursue early access programs despite recognizing the importance of discussing all treatment options with their patients (Bunnik & Aarts, 2021).

**Operational factors** included CUP experiences within the company, standardization, and global protocols, with enough product supply and the speed of the assessment by the NCA compared to the alternative of NPU. The interviewees indicated that the likelihood of pursuing CUPs increased when a company had prior CUP experience. Conversely, without this experience, NPU was often perceived as the more straightforward option. The variation in regulations for CUPs and NPU across EU member states may contribute to this confusion, with half of the interviewees expressing uncertainty about the distinction between the two. The plethora of terminologies used, including early access programs, CUP, and NPU, among others, was perceived as particularly confusing. Kimberly et al. (2017) concurred in underscoring the confusion stemming from the varied terminology while highlighting potential challenges, especially in an international context.

Comparisons with NPU frequently arose when considering factors influencing the decision to pursue a CUP. While both programs share similarities, the main difference is their benefit-risk assessment process and the length of the approval process. In the Netherlands, CUPs require a comprehensive benefit-risk assessment, whereas NPU relies on a physician’s individualized assessment based on the sponsor’s publication of study results. Thus, NPU may be considered earlier in the drug development process, potentially even before a CUP is considered or in parallel to an ongoing clinical trial. A CUP is typically applied in parallel with the marketing authorization application phase. The approval process may take weeks compared to only a few days for an urgent NPU request approval process. Thus, to enhance the familiarity and utility of CUPs, we emphasize the need for clear guidelines and information about their purpose and use while clearly outlining the differences between a CUP and NPU, with accessible information (e.g. Frequently Asked Questions on the NCA website to guide the decision between a CUP versus NPU) provided in a collective effort by the NCA(s) responsible for CUP and NPU approval. Furthermore, the benefit-risk assessment for CUP applications should be accelerated, as the current six-week assessment period is too lengthy compared to the significantly shorter approval times for NPU.

Notably, interactions with medical research ethics committees were not identified as an operational factor in our interview study. This is to be expected, as CUPs in the Netherlands are not subject to review by such a committee, in contrast to countries such as Italy, Spain, or the United States, where ethics approval is required (Borysowski et al., 2017). Additionally, CUPs are often initiated following clinical trials that have already secured full ethical approval and incorporated patient informed consent. This difference in the involvement of medical research ethics committees highlights the diversity of the regulatory landscape surrounding CUPs.

**Financial factors** in CUP implementation included the discontinuation by some pharmaceutical companies of free-of-charge medicine supply, as well as the uncertainty surrounding future reimbursement. Our findings show that such discontinuations often stem from financial constraints and the lack of reimbursement guarantees, which in turn limit patient access. From the perspective of healthcare systems, CUPs may offer notable financial benefits. Jommi et al. (2021) emphasized that CUPs can reduce costs for healthcare providers, and Dell’Anno et al. (2024) further demonstrated that CUPs can generate significant savings for the system as a whole. However, these benefits may come at a financial cost to the pharmaceutical companies supplying the medicines.

Upcoming legislative changes by the European Commission (EC) are expected to impact the future of CUPs (European Commission, 2023a, 2023b). The draft Regulation by the EC, published in April 2023, proposes expanding the criteria for CUPs by also including new therapeutic uses of authorized medicines in Article 26 (European Commission, 2023a). This shift could significantly broaden the scope of CUP and enable increased patient access to treatments that address unmet medical needs. Moreover, the emphasis on real-world data from CUPs is also reflected in the draft regulation, which highlights the importance of allowing for data on CUPs to be collected to inform decisions. On 4 June 2025, the Council of the EU adopted its position on the proposal to revise the EU’s general pharmaceutical legislation. Negotiations with the European Parliament to reach a final version of the new EU pharmaceutical legislation are currently ongoing and are expected to continue until at least the end of 2025. If implemented, these changes could enhance the utility of CUPs.

A limitation of this study was its primary focus on the Netherlands, which might not have fully captured the diversity of the regulatory landscape. Indeed, variations between EU member states could influence how pharmaceutical companies approach expanded access programs (e.g. CUPs). Despite this limitation, the factors identified in this study provide a foundational basis for future research to explore the motives of pharmaceutical companies toward CUPs. A second limitation concerns the relatively small number of interviewees (n = 10). However, all participants were experts in the regulatory and medical field, and no new themes emerged during the final interviews, suggesting that data saturation was likely reached. This conclusion was further supported by a follow-up presentation to members of the Dutch Association Innovative Medicines on 21 November 2023, during which no additional insights were identified. While the sample size aligns with qualitative research standards, especially in homogeneous expert groups where saturation typically occurs within 9–17 interviews (Hennink & Kauser, 2022), it may still have limited the diversity of perspectives captured. Conducting a more comprehensive analysis that includes a wider range of EU member states would provide valuable insights into the differences and similarities in CUPs while facilitating improved and harmonized CUP regulations across Europe.

## 5. Conclusion

In conclusion, early access to treatments is crucial for patients suffering from serious diseases. From the patient’s perspective, the specific regulatory framework may be less important than timely access to necessary medicines. This study indicates that the lack of clarity within the CUP framework may discourage companies from making their medicines available as early as possible.

Aligning the CUP framework with regulations on data collection during a CUP or NPU could motivate more companies to apply for CUPs and provide valuable insights into the real-world use of these medicines. Most importantly, this alignment may benefit patients by facilitating earlier access to medicines.

## Authors’ contributions

Aimée Timmerman: Writing – review & editing, Writing – original draft, Visualization, Formal analysis, Methodology, Conceptualization. Nienke Rodenhuis: Writing – review & editing, Validation. Lucia Marie Albertine Crane-van Opstal: Writing – review & editing, Validation. Anthonius de Boer: Writing – review & editing, Validation. Leon Bongers: Writing – review & editing, Formal analysis, Methodology, Conceptualization, Supervision, Validation. Anna Maria Gerdina Pasmooij: Writing – review & editing, Formal analysis, Methodology, Conceptualization, Supervision, Validation.

## Ethics approval and consent to participate

Ethical approval was obtained from the Research Ethics Committee (CETO) of the Faculty of Arts, University of Groningen with protocol number ID 102394219. All participants signed an informed consent form.

## Supplementary Material

Supplementary Material.docx

## Data Availability

The data that support the findings of this study are available on request from the corresponding author. The data are not publicly available due to privacy restrictions.
