# Supplementary material for: Factors influencing pharmaceutical companies’ decisions to pursue compassionate use programs in the EU: a qualitative study in The Netherlands
Source: J Pharm Policy Pract. 2026 Jan 6;19(1):2605391. doi: 10.1080/20523211.2025.2605391 (PMC12777770; doi:10.1080/20523211.2025.2605391)
Supplement: Supplementary Material.docx [file JPPP_A_2605391_SM1751.docx]

**Interview Guide**

**[Introduction]**

Good day, my name is Aimée, and first of all, I would like to sincerely thank you for participating in this interview. I am a master's student at the University of Groningen, and this research for the MEB is part of my graduation project. The goal of my research is to map the motivations of companies for applying for a CUP.

**[Purpose of the Interview]**

It is very valuable to have the opportunity to interview you. The purpose of this interview is to gather information on the factors that influence the decision to apply for a CUP with the MEB. This will include general factors as well as factors specific to products for which a CUP was or was not requested.

**[Consent]**

Before we begin the interview, I want to make sure that you have read, understood, and signed the informed consent form that we sent to you by email a few days ago. Do you have any questions about it? If anything is unclear, I will briefly go over the following points:

- We will anonymize your answers after the interview so that they cannot be traced back to a specific product or company.
- You may stop the interview at any time.
- You can request that your data will be deleted before it is anonymized.
- Do you have any questions regarding this information?
- Do you agree with the procedures mentioned above?
- Finally, we would like to record this interview. Do you consent to that?

**[Background Information and Knowledge about CUP]**

1. Could you briefly introduce yourself? Who are you, what is your role, and how long have you been in this position?
2. Do you have experience with the CUP policy of the MEB?

**[CUP Decision-Making within the Company]**

I will start with some general questions about CUP decision-making within your company. I will go into more detail later on specific factors that might play a role. *Please indicate whether your company has ever applied for a CUP, yes or no.*

1. Can you tell me about the decision-making process for a CUP within your company? Is there a protocol or SOP (Standard Operating Procedure) for this? Is it standard to consider a CUP during product development?
   a. Does this vary by therapeutic area, or is it the same across the company? Are there specific departments involved?
   b. At what point in the drug development process is a CUP considered within your company, and why?
   c. Can you tell me about the decision-making process regarding the funding of a CUP?
   d. How does this relate to health insurers?
2. Can you tell me which factors play a role in your company’s decision to consider a CUP? What are the motivating factors, and what are the barriers? Can you name the three most influential factors? *Why?*
   a. What are the advantages (benefits) for your company in offering a CUP? *And why?*
   b. What are the disadvantages (challenges) for your company in offering a CUP? *And why?*
3. In addition to the CUP, are there any other programs or initiatives your company uses to provide access to medicines that are not yet registered?
   a. What is your company’s role in providing NPU for this?
   b. Does your company have a preferred route for getting an unregistered product to patients? *Why?*
4. Can you tell me about the process of applying for a CUP in the Netherlands compared to other European countries?
   a. What are the advantages and disadvantages of applying for a CUP in the Netherlands compared to other European countries? *Can you elaborate?*
   b. What factors influence the decision to offer a CUP in one country versus multiple countries? *Which factor carries the most weight?*
5. Are there things you think the MEB could do to make a CUP application more attractive to companies?

**[Approved CUPs]**

In the past, your company has provided a CUP for [product name(s)], and I would like to ask some specific questions about this.

1. What were the reasons for applying for a CUP for this product? Can you elaborate?
   a. *Which reason had the biggest impact? Can you list the top three factors that had the greatest impact? (This will depend on how the conversation develops).*
   b. Were there any doubts?
   c. How was the decision made?
   d. Do you monitor safety and efficacy differently for a CUP compared to a registered product? *If yes, what are these differences and why?*
2. How did the CUP application process with the MEB go?
   a. How would you rate the available information on the MEB’s website regarding the CUP application?
   b. What was clear?
   c. What was unclear?
   d. What aspects of the application process were challenging? *Why?*
3. Once the marketing authorization was granted for this product, did patients continue to receive the product until it was reimbursed by the health insurance?
   a. What was your company’s role in this process?
   b. Who financed the medication until it was reimbursed?
4. Could you comment on the awareness and acceptance of the product because of the CUP?
5. Would you consider providing a CUP again for this product?

**[Products for which a CUP was not Requested]**

For [product name(s)], your company did not request a CUP, even though this product could have qualified. I would like to ask you some questions about this.

1. Did your company consider applying for a CUP for this product? *Can you elaborate?*
2. What were the main reasons for not applying for a CUP?
   a. Can you explain this in more detail? *Feel free to go through the factors mentioned in question 4. Can you list the top 3 reasons that had the most influence on this decision?*
3. Has your company implemented other initiatives or programs to provide access to this product before it was registered, such as NPU?
   a. What role did your company play in this process?
   16. Did your company apply for a CUP for this product in another European country? *Why or why not?*

**[Closing]**

Your answers have been very helpful, thank you for that. Those were all my questions. Is there anything else you would like to add, any perspectives or opinions you would like to share? If not, I would like to thank you for your time and the interview.
